# The First Record of *Monochamus saltuarius* (Coleoptera; Cerambycidae) as Vector of *Bursaphelenchus xylophilus* and Its New Potential Hosts in China

**DOI:** 10.3390/insects11090636

**Published:** 2020-09-16

**Authors:** Min Li, Huan Li, Ruo-Cheng Sheng, Hui Sun, Shou-Hui Sun, Feng-Mao Chen

**Affiliations:** 1 Co-Innovation Center for the Sustainable Forestry in Southern China, College of Forestry, Nanjing Forestry University, Nanjing 210037, China; lm950330@njfu.edu.cn (M.L.); lhuan@njfu.edu.cn (H.L.); rcsheng@njfu.edu.cn (R.-C.S.); hui.sun@njfu.edu.cn (H.S.); 2 College of Forestry, Shenyang Agricultural University, Shenyang 110866, China; sysshh@163.com

**Keywords:** conifer tree, pinewood nematode, pine sawyer, host plants

## Abstract

**Simple Summary:**

During a survey of pine wilt disease in 2017, thousands of dead pitch pines (*Pinus koraiensis*) were discovered in Fengcheng, Liaoning Province, China. Samples were taken from several regions of the bole, and *Bursaphelenchus xylophilus* was collected from the dead pitch pine samples. *Monochamus saltuarius* is an indigenous insect in this area, but there is no experimental evidence that *M. saltuarius* can transport *B. xylophilus* in China. The experimental results showed that *M. saltuarius* was the new vector of pine wilt disease in China. In laboratory experiments, *M. saltuarius* fed and transmitted *B. xylophilus* not only on pines but also on other conifers. Taxus, Juniperus, Sabina and Podocarpus are new tree species upon which *B. xylophilus* can be fed and transmitted by *M. saltuarius*. *Picea pungens*, *Picea asperata*, *Abies fabri*, *A. holophylla*, *Larix principis-rupprechtii*, *L. olgensis*, *Juniperus formosana*, *Sabina chinensis* and *Podocarpus macrophyllus* are new species of coniferous trees known to be able to be infected by *B. xylophilus* in a laboratory setting. This study raises awareness to prevent the disease from expanding and provides a reference for the control of pine wilt disease.

**Abstract:**

Pine wilt disease was first discovered in Dongtang town, Liaoning Province, China, in 2017. However, no record of *Monochamus alteratus* existed in Fengcheng, where *M. saltuarius* is an indigenous insect, and no experimental evidence has thus far indicated that *M. saltuarius* can transport the *Bursaphelenchus xylophilus* in China. In this study, we investigated whether *M. saltuarius* is a vector of *B. xylophilus* in China. On the sixth day after eclosion, beetles began to transmit nematodes into the twigs. The transmission period of nematodes is known to be able to last for 48 days after beetle emergence. In laboratory experiments, *M. saltuarius* fed and transmitted *B. xylophilus* not only on pines but also on other non-*Pinus* conifers. The non-*Pinus* conifers preferred by *M. saltuarius* for feeding are *Picea pungens*, *Picea asperata*, and *Abies fabri*. The experimental results show that *M. saltuarius* functions as a vector of *B. xylophilus* in northeast China.

## 1. Introduction

Pine wilt disease (PWD) is caused by the pinewood nematode (PWN) *Bursaphelenchus xylophilus* (Steiner and Buhrer, 1934) Nickle, 1970. With the development of economic globalization and increases in human migration, many nonnative organisms have been introduced into various ecological communities [1]. In many cases, when no natural enemy is present and the living environment is favorable, exotic organisms are likely to cause considerable harm to the ecosystem into which they enter. The PWN is native to north America [1], and this nematode has caused serious pine disease after its introduction to new regions, especially in East Asian countries such as Japan [2], China [3] and Korea [4]. In China, the first case of *Pinus thunbergii* Parl. showing PWD and withering was found in Zijin Mountain, Nanjing, Jiangsu Province, in 1982 [5]. Despite many measures being implemented during the past four decades, PWD has spread quickly to other provinces, and an optimal treatment has not yet been developed for this disease [6].

Insects related to PWD worldwide include 21 species of Cerambycidae, 1 species of Buprestidae and 2 species of Curculionidae, but the main vectors of PWD are beetles of the genus *Monochamus* (Cerambycidae) [7,8]. To date, 13 beetles of the genus *Monochamus* have been shown to carry PWNs. In north America, *Monochamus carolinesis* Olivier [9], *M. scutellatus* Say [10], *M. titillator* Fabricius [11], *M. obtusus* Casey [12], *M. notatus* Drury [10], *M. marmorator* Kirby and *M. mutator* LeConte [13] are vectors of PWN. In Asia, *M. alternatus* Hope [14], *M. saltuarius* Gebler [15] and *M. nitens* Bates can carry and transfer PWNs. Throughout Europe, *M. galloprovincialis* Olivier is the only species that currently functions as a vector of PWNs [16], while *M. sutor* Linné [17] and *M. urussovi* Fischer-Waldheim [18] are also potential vectors of PWN. In Europe and North Africa, *M. galloprovincialis* [19,20] has been reported to be the local PWN vector.

During a survey of PWD in 2017, thousands of dead pitch pines (*Pinus koraiensis* Sieb. et Zucc. Fl.) were discovered in Fengcheng, Liaoning Province and attracted the attention of the National Forestry and Grassland Administration of China [21]. In a 2017 trap survey, we only found *M. saltuarius*, a pine tree beetle. A large number of such beetles had colonized weakened and dying pine trees in Dongtang town, which is the epidemic area in Fengcheng. *Monochamus saltuarius* belongs to the family Cerambycidae, and the species has been found to carry PWNs under their elytra [22]. This scenario has been shown to be widespread in Japan and South Korea, and the species has been reported to carry and transmit PWNs [23,24]. However, this beetle is an indigenous insect in this area [25], and to our knowledge, no reports have indicated that *M. saltuarius* transmits PWNs in China.

The PWN is generally believed to be transmitted through timber, mainly by beetles emerging from PWN-infected trees. In Fengcheng, the timber industry generates a large revenue and provides employment opportunities for thousands of people. Thus, this disease has caused serious social and economic impacts in this area. *Pinus koraiensis* is one of the most important pine species in this area, but unfortunately, this pine is also the preferred host of PWN [26]. Although many studies have been conducted in the past century, no effective strategy has been found to manage PWD. In addition, other genera, such as *Abies*, *Cedrus, Chamaecyparis*, *Larix*, *Pseudotesuga* and *Picea*, are also considered suitable PWN hosts [27]. Many types of trees exist on the outskirts of Dongtang town in addition to *P. koraiensis* [26], *P. tabuliformis* Carr [28] and *P. armandii* Franch [29], which have been shown to be infested by PWNs; the city also hosts *Larix principis-rupprechtii* Mayr, *L. olgensis* Henry, *Taxus chinensis* (Pilger) Rehd, *Picea pungens*, *Picea asperata* Mast, *Juniperus formosana* Hayata, *Sabina chinensis* (L.) Ant, *Abies fabri* (Mast.) Craib, *A. holophylla* Maxim and *Podocarpus macrophyllus* (Thunb.) D. Don, which account for large proportions and are the main conifer species.

The purpose of this work was to (I) assess whether *M. saltuarius* can carry PWNs; (II) confirm whether nematodes can be transmitted by feeding; and (III) test whether *M. saltuarius* can feed on conifer trees other than pine trees and whether these tree species can be infected by PWNs.

## 2. Materials and Methods

### 2.1. Source and Preservation of Dead Trees

During the period from September to December 2017, 1200 *P. koraiensis* trees (25–30 years old) that had been killed by PWNs were felled in the Dongtang town region of Fengcheng, Liaoning Province, China. Thirty trees were cut into logs (1.0–1.2 m- in length) and transported to the Forest Pest Control and Quarantine Bureau of Fengcheng City where they were placed outdoors. Thereafter, the timber was kept in a top-covered shed field cage (200 × 250 × 200 cm) with sidewalls composed of 0.2-cm metal mesh. The logs were watered every 3 days until the end of the observation period, and daily observations were recorded from early May to the end of August 2018.

### 2.2. Experimental Conifers and Food Sources

The following mature trees were obtained from the Fengcheng Nursery (Fengcheng, Liaoning Province; 40°28′7′′ N, 124°03′18′′ W) and kept in an open-air location: 5-year-old *P. koraiensis* seedlings; 18-year-old *L. principis*, *L. olgensis* and *T. chinensis* seedlings; and 20-year-old *Picea pungens*, *Picea asperata*, *J. formosana*, *S. chinensis*, *A. fabri*, *A. holophylla* and *Podocarpus macrophyllus* seedlings. All plant material was collected from this nursery, and twigs used for beetle food were recut to lengths of 15 cm in the laboratory.

### 2.3. Detection of PWN Carried by M. saltuarius

As the beetles emerged, they were captured in a cage from the Forest Pest Control and Quarantine Bureau and sexed, and the surrounding temperature and humidity were recorded daily. The number of dispersed juveniles carried by each newly emerged beetle was estimated by a non-destructive method, i.e., stereomicroscope examination of both the first abdominal and metathoracic spiracular openings to determine whether or not they contained PWN [30]. Sixteen beetles were selected and sectioned after examination and any remaining nematodes in the adults were extracted by the Baermann funnel technique, which is, according to Shuo [31], the most efficient method by which to extract nematodes from a vector body. Then, approximately 13 mL of liquid was collected at the bottom of the funnel, and the nematodes were collected. The next step was to shake the sample well and count the number of nematodes under an inverted light microscope (Carl Zeiss Imager M2, 37,081 Gattingen, Germany). Three replications were conducted for each isolate.

For further verification, DNA was extracted from individual nematodes. The internal transcribed spacer (ITS) primers F194 (5′-CGTAACAAGGTAGCTGTAG-3′) and 5368r (5′-TCCTCCGCTAAATGATATG-3′) were used to identify the nematodes [32,33]. The following reagents were required for this identification: protease K, 10x PCR buffer, 10x Ex*Taq* buffer, and dNTP mixture, which were purchased from Takara Biomedical Technology Co., Ltd. (Beijing, China); 2x Easy*Taq* PCR SuperMix, which was purchased from Genscript Biomedical Technology Co., Ltd. (Nanjing, China); and MgCl_2_ and primers, which were synthesized by Sangon Biomedical Technology Co., Ltd. (Shanghai, China).

Nematodes extracted from beetles were selected under a Leica DM500 microscope and washed twice with ddH_2_O. A total of 10 nematodes were randomly selected for molecular identification. The nematodes were placed in a 1.5-mL centrifuge tube, and each centrifuge tube had one nematode with 8 μL of nematode lysis buffer and 2 μL of protease K. The samples were incubated at 65 °C for 35 min, heated to 95 °C for 7 min, and centrifuged for 10 s to extract DNA. The total volume of the polymerase chain reaction (PCR) was 25 μL, including 2.5 μL of 10× PCR buffer, 2 μL of 2.5 mmol/L MgCl_2_, 1 μL of 10× Ex*Taq* buffer, 1 μL of each primer, 1.5 μL of template DNA (supernatant from the 1.5-mL centrifuge tube) and 14.8 μL of ddH_2_O [34]. The amplification instrument was a PTC-200 DNA Engine (Hangzhou Bioer Technology Co. Ltd. Hangzhou, China). The following protocol was used for PCR amplification of the rDNA-ITS region: initial denaturation at 94 °C for 5 min, 40 cycles of 94 °C for 50 s, 49 °C for 40 s and 72 °C for 1 min; and a final extension period at 72 °C for 7 min [35]. The PCR products of nematodes were further DNA sequenced by Jie Li Biomedical Technology Co., Ltd. (Shanghai, China).

### 2.4. Experiments on Feeding Transmission by M. saltuarius

One hundred and ten newly emerged adults were transferred into individual cages (30 × 40 × 50 cm) constructed of 0.2-cm wire mesh, provided with fresh twigs, and kept at room temperature. Fifty beetles were provided *P. koraiensis* twigs as food and the other 60 beetles were provided non-*Pinus* conifer twigs as food (6 beetles per tree species). To prepare beetle food for the experiments, fresh twigs were clipped from *P. koraiensis*, *L. principis*, *L. olgensis*, *T. chinensis*, *Picea pungens*, *Picea asperata*, *J. formosana*, *S. chinensis*, *A. fabri*, *A. holophylla* and *Podocarpus macrophyllus* (including the new annual part and the two-year-old part) and cut into sections (15-cm in length). Each twig section was washed with running tap water and placed in the experimental cage as the food source for the beetle. Each twig was separately placed in three cages for repetition. New twigs were placed in the cages every 3 days until the beetles died. The 3-day feeding area (where the bark had been chewed) on each non-*Pinus* conifer twig was measured using transparent square millimeter paper [36] and the replaced twigs were extracted by the Baermann funnel technique. The amount of non-*Pinus* conifer bark consumed (mm^2^) was used as the response variable for the analysis of feeding preference. The number of PWNs in a beetle was the sum of the number of PWNs transmitted into the twigs and the number of PWNs isolated after the beetle was dead.

To further verify the ability of the beetle to transmit PWNs, ten 5-year-old *P. koraiensis* potted seedlings (approximately 110 cm tall) without disease were selected on July 4, and these trees were placed in two experimental cages (250 cm × 250 cm × 270 cm). One cage contained 15 beetles, and the other cage without beetles was used as a control. The seedlings were examined weekly for disease symptoms, which included wilting leaf discoloration and leaf fall. After the seedlings died, stem sections were cut from seedlings at each of the following positions: upper part (main shoot), middle part, lower part (above ground level), needles, branches and roots. Stem samples were sliced thin and placed on Baermann funnels to extract nematodes.

### 2.5. Data Analysis

One-way analysis of variance (ANOVA) was used to determine the significance of the mean differences in the number of nematodes separated from beetles. GraphPad Prism (8.0.1 for Windows) was used for chart construction.

## 3. Results

### 3.1. Emergence of M. saltuarius and Identification of Nematodes

**Figure 1:**
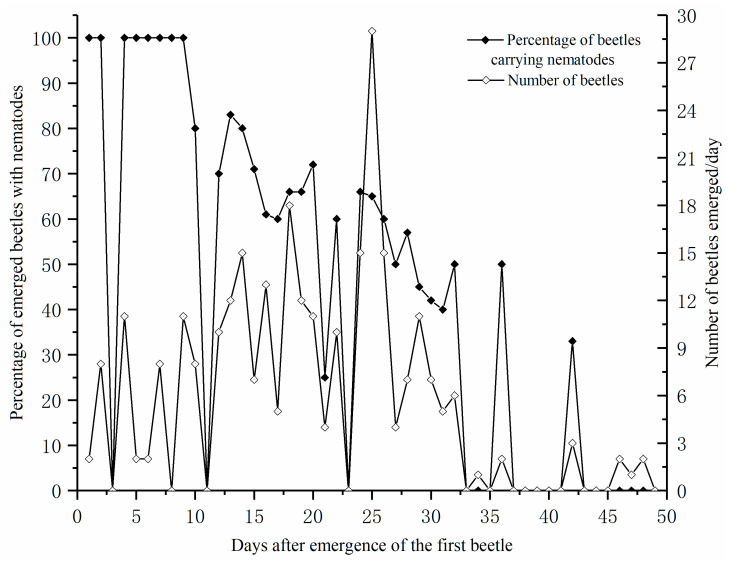
The percentage of beetles carrying nematodes (◆) and the emergence of *Monochamus saltuarius* from pine timbers (◇) during the emergence period in 2018.

The first *M. saltuarius* beetle emerged from the *P. koraiensis* timbers in the cage on May 9, and the full emergence period of the beetles lasted 49 days. During this period in 2018, the average temperature and humidity were 19 °C (11.0–25.0 °C) and 60.0% (30.0–90.0%), respectively. We collected 279 adults, and their sex ratio was almost balanced (1.08:1; 145 males and 134 females). During the initial 5 days, only a few beetles emerged. However, nematode-carrying beetles accounted for a high proportion of the total number of beetles that emerged (Figure 1), and this proportion decreased over time.

Sequences were obtained from 10 selected nematodes, and the product length of the rDNA-ITS region was 916 bp according to the sequencing and Blast search in NCBI GenBank, which was 99–100% similar to that of *B. xylophilus* (https://www.ncbi.nlm.nih.gov/nucleotide/MN006173.1/).

The average number of nematodes per beetle was 7970 ± 327. Eight female beetles carried an average of 8010 nematodes, while 8 male beetles carried an average of 7920 nematodes, and the numbers carried by females and males did not significantly differ. Based on the number of extracted nematodes, the numbers of nematodes carried by male and female *M. saltuarius* beetles were similar with no significant difference.

### 3.2. Types of Transmission of PWNs into Pine Twigs through M. saltuarius Feeding

The transmission of PWNs to fresh pine twigs as indicated by the number of nematodes carried by the beetles was studied in further detail. Some beetles died prematurely during the test, so some valid data were selected. The details for the transmission of nematodes by individual beetles are shown in Table 1. The transmission of nematodes started from the 6th day after adult emergence. The first batch of nematodes was extracted from the feeding wounds after the beetles emerged and started their maturity feeding (e.g., beetle 7). This condition proved that *M. saltuarius* can transmit PWNs by feeding.

During the entire feeding period, the maximum number of nematodes transmitted by a beetle to the twigs was 550 (beetle 9), and the maximum number of nematodes extracted from a dead beetle was 21,000 (beetle 4). The number of nematodes transmitted to branches ranged from 1 to 550 within three days.

### 3.3. Experiment of M. saltuarius Feeding on Potted Seedlings

In this experiment involving feeding on potted seedlings, two of the tested trees in the experimental cage with *M. saltuarius* died on August 31, and PWNs were detected. The others died on October 3 and October 26, and PWNs were also found in these dead trees. Among the four dead trees, PWNs were found in all the trunks and branches, as well as in the needles of three experimental trees (Nos. 1, 3 and 5). Three of the five experimental trees had PWNs in the roots (Nos. 1, 2 and 3). Conversely, none of the control trees died because these trees were placed in the experimental cage without beetles (Table 2).

### 3.4. Transmission of PWNs by Mature M. saltuarius Feeding on Non-Pinus Conifer Tree Species

In all 10 non-*Pinus* conifers, 60 beetles fed under compulsory feeding conditions and successfully transmitted PWNs. Among the 10 conifer species, the species preferred by *M. saltuarius* were *Picea pungens*, *Picea asperata* and *A. fabri*. The order of beetle feeding preference (as determined by comparing the size of the feeding area) was as follows: *Picea asperata* > *Picea pungens* > *A. fabri* > *A. holophylla* > *L. principis* > *J. formosana* > *L. olgensis* > *S. chinensis* > *Podocarpus macrophyllus* > *T. chinensis* (Table 3).

## 4. Discussion

In China, no research has been conducted on the transmission of PWNs by *M. saltuarius* because this species has not been reported to carry PWNs in mainland China. This vector of PWNs has received considerable attention since the outbreak of PWD in Fengcheng City in 2017. The above results showed that the young trees of *P. koraiensis* died due to PWD because they were fed on by *M. saltuarius* carrying PWNs in vivo. Under the climate conditions of Fengcheng City, PWNs can migrate from dead wood to *M. saltuarius* and break away from *M. saltuarius* during the feeding process to invade healthy pine trees. Therefore, our experiment showed for the first time that *M. saltuarius* beetles are carriers and disseminators of PWNs in China, thus providing a basis for the early detection and prevention of PWD in this area. On the sixth day after eclosion, the beetles began to transmit nematodes into the twigs. The transmission period of nematodes lasted up 48 days after beetle emergence. Tree No. 4 in the experimental cage with *M. saltuarius* showed little mechanical damage due to maturation feeding, which may explain why no PWNs specimens recovered (insufficient *B. xylophilus* invasion) [37]. The geographical environment and temperature differ substantially from southern to northern China. Cold stress may influence the expansion of PWD and the reproduction of *M. alternatus* in China [38]. Previous studies have shown that the areas suitable for the development of PWD are mainly in the southeast of China and the northernmost areas in the southern part of Beijing, Tianjin and southern Hebei [21,39]. However, PWD has been found in Liaoning Province, where *M. saltuarius* is distributed, but no *M. alternatus* have been found. According to previous studies, *M. alternatus* is a native sawyer beetle in China, although PWD did not occur and spread in China until 1982 [40], indicating that although *M. alternatus* can transmit PWN as a vector, there were no PWNs in mainland China at that time. Therefore, PWD had not occurred. *M. saltuarius* is also a native longhorn beetle in northern China. There are many possible reasons for the occurrence of PWNs in Fengcheng, Liaoning Province. The most likely scenario is that pine wood products infected with PWN in southern China were artificially transported to Fengcheng City, and *M. alternatus* then emerged from these trees and fed on the local Korean pine. However, *M. alternatus* could not survive the low winter temperatures (the annual average temperature is below 10 °C) and thus died [41]. However, PWNs can tolerate low temperatures [42] and survive until *M. saltuarius* eclosion in the following year, thus, expanding their range and dispersing. The alien PWN adopted the native *M. saltuarius* as a novel vector in this newly invaded area [43].

Northeast China is rich in tree species and conifers. Our experiments showed that *M. saltuarius* not only feeds on Korean pine but can also spread PWNs to other conifer trees. Moreover, *Taxus*, *Juniperus*, *Sabina* and *Podocarpus* trees are new species that can be fed on by *M. saltuarius*. *Picea pungens, Picea asperata*, *A. holophylla*, *L. principis-rupprechtii*, and *L. olgensis* are new species of conifer trees that can be infected with PWD. Interestingly, after approximately 20 min of feeding on *T. chinensis*, *M. saltuarius* slowed and even entered a state of pseudo death (shock), which may have been caused by the chemical composition of *T. chinensis*. The beetles then recovered after approximately half a day. After recovery, *M. saltuarius* continued feeding and then repeated the process. The PWNs successfully developed into adults in *T. chinensis* and were not affected. We urgently need to increase awareness regarding the prevention of PWD. *Pine massoniana* was originally considered the disease-resistant tree species when PWD first occurred in Nanjing, but the main pine species damaged by PWN in southeast China has recently changed from *P. thumbergii* to *P. massoniana* [44].

## 5. Conclusions

The present study showed that *M. saltuarius* was a vector of PWD in northeast China. On the sixth day after eclosion, the beetle began to transmit nematodes into the twigs. The transmission period of nematodes lasted up 48 days after beetle emergence. *M. saltuarius* not only feeds on pines, but also spreads PWN to other conifers. The tree species preferred by *M. saltuarius* for feeding are *P. koraiensis*, *Picea pungens*, *Picea asperata*, and *Abies fabri*. Currently, PWNs have spread to Liaoning Province, reflecting the inevitability that PWNs will be detected in Jilin Province, Heilongjiang Province and other high-latitude regions in China. It is important to raise awareness in order to prevent the disease from expanding and to provide a reference for the control of PWD.

## Figures and Tables

**Table 1:** Summary of *Bursaphelenchus xylophilus* transmission by several *Monochamus saltuarius* individuals feeding on twigs of *Pinus koraiensis* during the entire feeding period.

Code of Beetles	First Detection of Nematodes on Twig (Days after Beetle Emergence)	Total Number of Nematodes Transmitted to Twig	Number of Nematodes Retained in Dead Beetle	Number of Nematodes Transmitted Per Day (Max-Min)	Last Detection of Nematodes (Days after Beetle Emergence)	Number of Days of Nematode Presence in the Feeding Wound during the Entire Feeding Period
1	12	49	0	21–1	48	21
2	18	64	0	50–2	48	18
3	9	100	5	43–1	27	12
4	9	118	21,000	116–2	21	12
5	15	63	33	38–1	48	21
6	12	44	586	23–1	24	18
7	6	69	0	22–1	33	21
8	9	69	3450	27–1	36	9
9	15	550	0	550–550	9	12
10	15	20	0	9–1	24	27

**Table 2:** Number of *Bursaphelenchus xylophilus* isolated from treated trees in the experimental cage. Data represent the means ± SD of three replicates.

Treatment	No.	Date of Death	Isolation of *B. xylophilus* from:					
			Needles	Upper Stems	Middle Stems	Lower Stems	Branches	Roots
Treated trees in the experimental cage (with *Monochamus saltuarius*)	1	August 31	573 ± 130	13,250 ± 2300	8760 ± 1750	8890 ± 1230	15,340 ± 4430	130 ± 75
	2	October 3	0	0	3200 ± 550	2250 ± 300	3595 ± 376	63 ± 10
	3	August 31	330 ± 80	17,275 ± 5310	13,381 ± 3333	14,750 ± 2788	25,627 ± 8358	5112 ± 58
	4	/	/	/	/	/	/	/
	5	October 26	119 ± 27	810 ± 120	573 ± 50	0	1139 ± 298	0
Treated trees in the experimental cage (without *Monochamus saltuarius*)	1	/	/	/	/	/	/	/
	2	/	/	/	/	/	/	/
	3	/	/	/	/	/	/	/
	4	/	/	/	/	/	/	/
	5	/	/	/	/	/	/	/

**Table 3:** Bark area consumed by *Monochamus saltuarius* and the number of *Bursaphelenchus xylophilus* entering twigs in 3 days. Data represent the means ± SD of three replicates. Means in the column followed by the same letter did not differ significantly at *p* < 0.05 (method of multiple comparisons).

Tree Species	Bark Area Consumed/3 Days (mm^2^)	Number of *Bursaphelenchus xylophilus* Entering Twigs in 3 Days
Picea asperata *	(694 ± 7) a	(11 ± 1) a
Picea pungens *	(650 ± 12) a	(17 ± 3) a
Abies fabri *	(517 ± 15) a	(13 ± 5) a
Abies holophylla *	(444 ± 10) a	(11 ± 2) a
*Juniperus formosana*	(316 ± 7) b	(11 ± 3) a
*Sabina chinensis*	(308 ± 5) b	(16 ± 1) a
*Larix principis*	(335 ± 20) b	(11 ± 9) a
*Larix olgensis*	(314 ± 18) b	(17 ± 1) a
*Podocarpus macrophyllus* ^▲^	(205 ± 4) c	(13 ± 2) a
*Taxus chinensis* ^▲^	(139 ± 3) c	(9 ± 6) a
*F*	736.6	1.421
*P*	<0.0001	0.244

*: The favorite species of *Monochamus saltuarius* among the ten conifer species. ^▲^: The unpalatable species for *Monochamus saltuarius* among the ten conifer species.
